# Supplementary material for: Low threshold and efficient multiple exciton generation in halide perovskite nanocrystals
Source: Nat Commun. 2018 Oct 10;9:4197. doi: 10.1038/s41467-018-06596-1 (PMC6180109; doi:10.1038/s41467-018-06596-1)
Supplement: Supplementary file 1 — Supplementary Information [file 41467_2018_6596_MOESM1_ESM.docx]

Supplementary Information for

**Low Threshold and Efficient Multiple Exciton Generation**

**in Halide Perovskite Nanocrystals**

Mingjie Li^1#^, Raihana Begum^2#^, Jianhui Fu^1^, Qiang Xu^1^, Teck Ming Koh^2^, Sjoerd A Veldhuis,^2^ Michael Grätzel^3^, Nripan Mathews^2,4^, Subodh Mhaisalkar^2,4^*, and Tze Chien Sum^1^*

^1^School of Physical and Mathematical Sciences, Nanyang Technological University, 21 Nanyang Link, Singapore 637371.

^2^Energy Research Institute @ NTU (ERI@N), 50 Nanyang Drive, Research Techno Plaza, X-Frontier Block, Level 5, Singapore 637553

^3^Laboratory of Photonics and Interfaces, Department of Chemistry and Chemical Engineering, Swiss Federal Institute of Technology, Station 6, CH-1015 Lausanne, Switzerland.

^4^School of Materials Science and Engineering, Nanyang Technological University, 50 Nanyang Avenue, Singapore 639798

*Correspondence to: [Tzechien@ntu.edu.sg](mailto:Tzechien@ntu.edu.sg); [Subodh@ntu.edu.sg](mailto:Subodh@ntu.edu.sg)

**
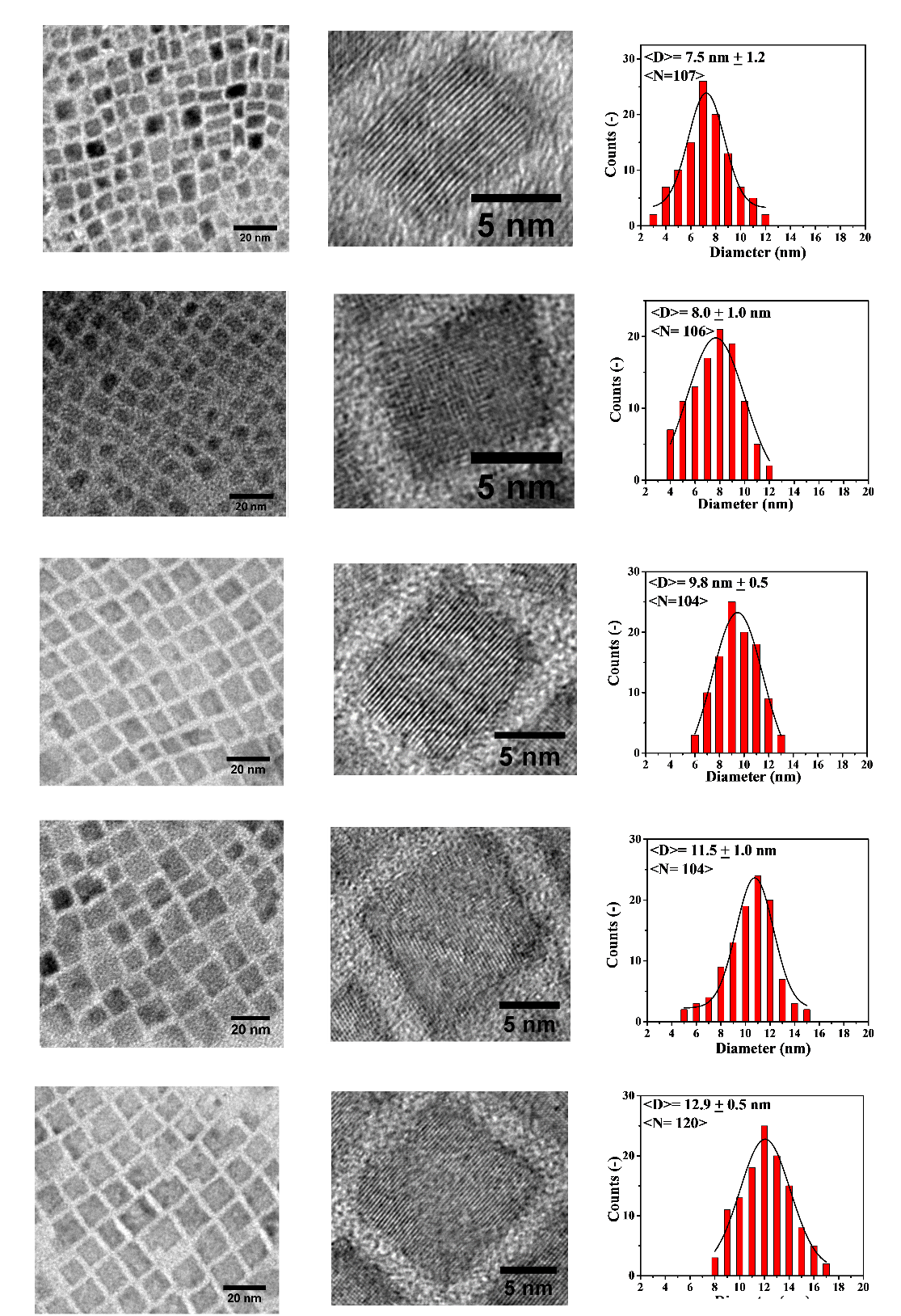
**

**Supplementary Figure 1** TEM images (left and middle panels) and particle size distributions (right panel) of FAPbI_3_ NCs with five different edge lengths. Scale bar: 20 nm (left panel), 5 nm (middle panel).

**Supplementary Figure 2** XRD patterns of FAPbI_3_ NCs with average edge length of (a) 12.9 nm, (b) 11.5 nm, (c) 9.8 nm, (d) 8.0 nm and (e) 7.5 nm.


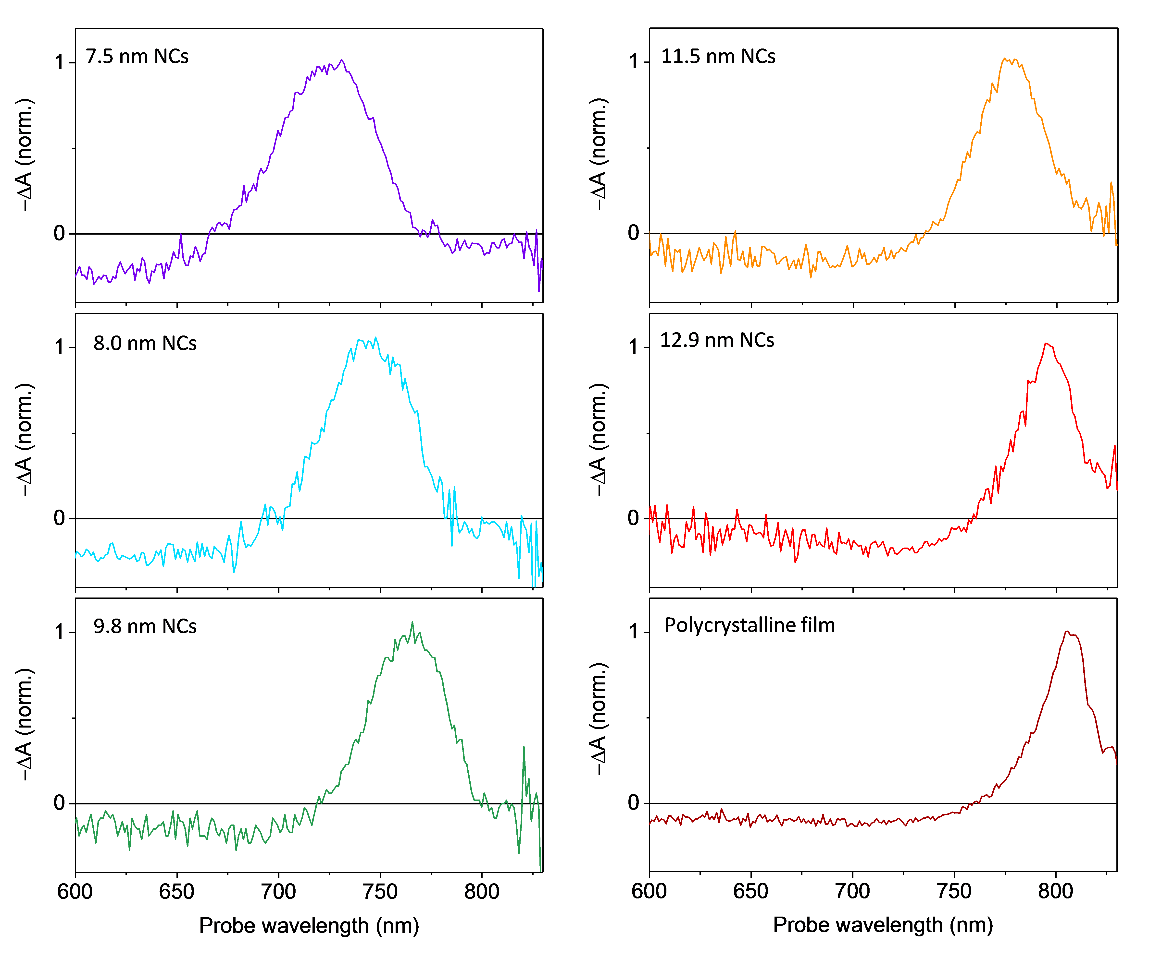


**Supplementary Figure 3** Normalized TA spectra of FAPbI_3_ NCs with different sizes and bulk-film counterpart at 1-2ps delay time under 400 nm excitation. The bandgap energies of NCs were taken from the PB peak positions.


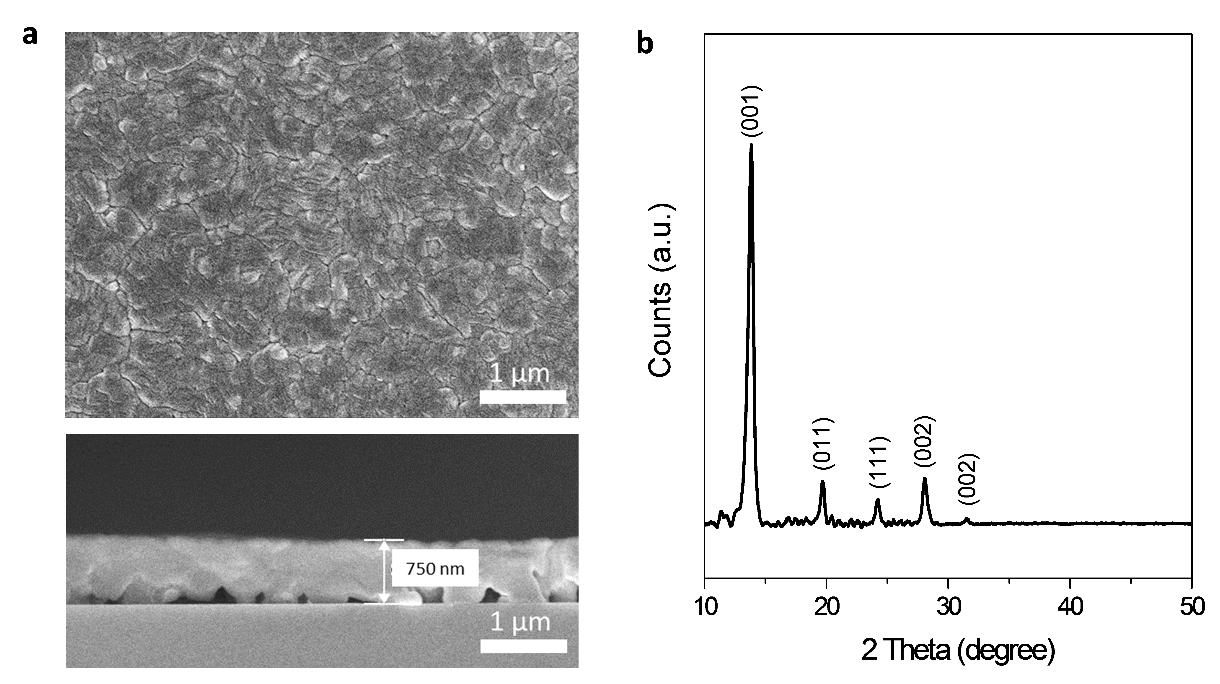


**Supplementary Figure 4** (a) Top-view (upper figure) and cross-sectional view (lower figure) of SEM images of FAPbI_3_ film. (b) XRD pattern of FAPbI_3_ film.


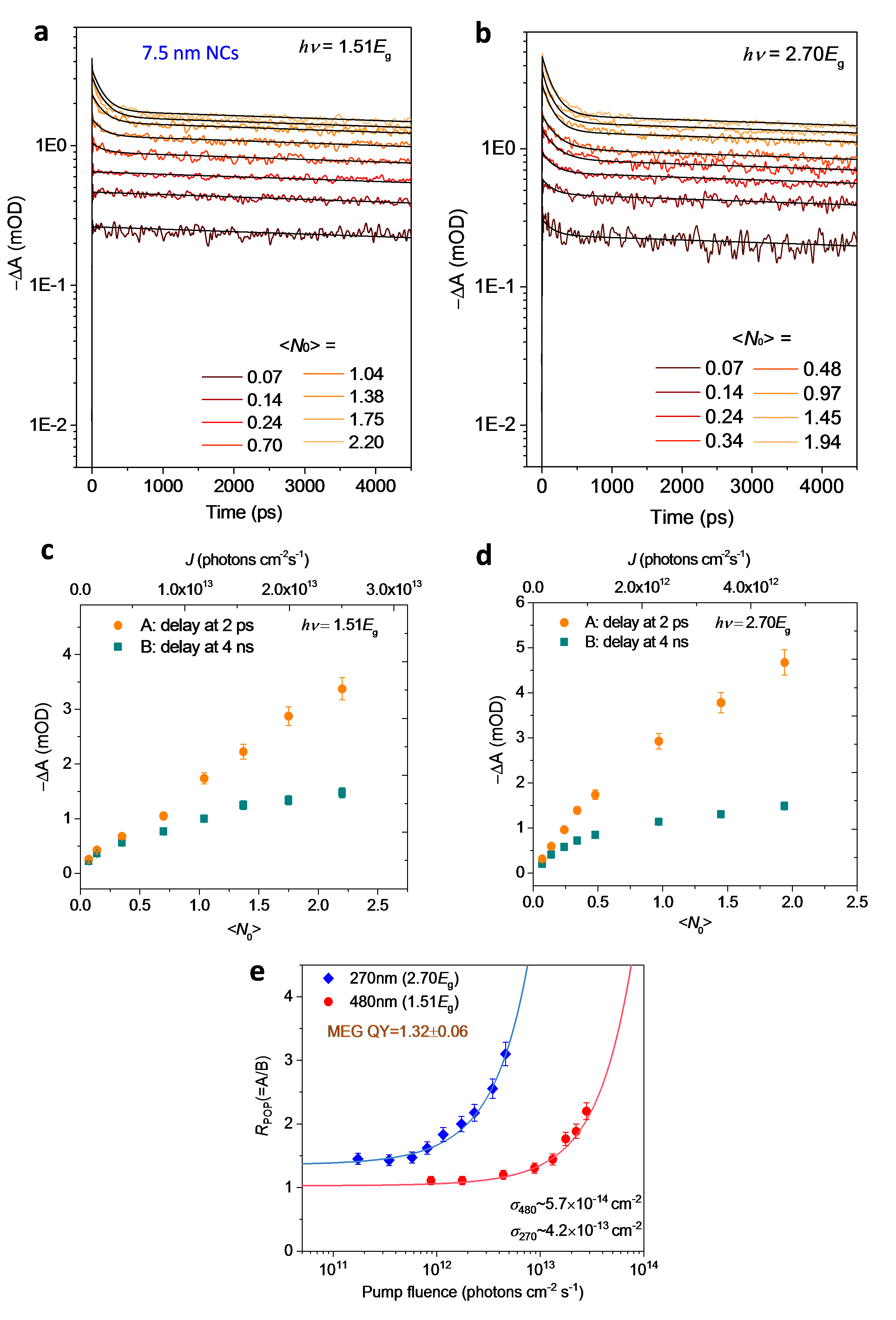


**Supplementary Figure 5** Band-edge PB dynamics at different <*N*_0_> generated by photon energies of (a) 1.51*E*g and (b) 2.70*E*g for 7.5-nm sized FAPbI_3_ NCs. Solid black lines are bi-exponential fittings with the fast decay of 160 ± 10 ps for biexciton lifetime. (c)-(d) TA amplitudes at delay times of A(Δt ~ 2ps) and B(Δt ~ 4ns) under different photoexcitations. (e) *R*_POP_ (=A/B) as a function of pump fluence.


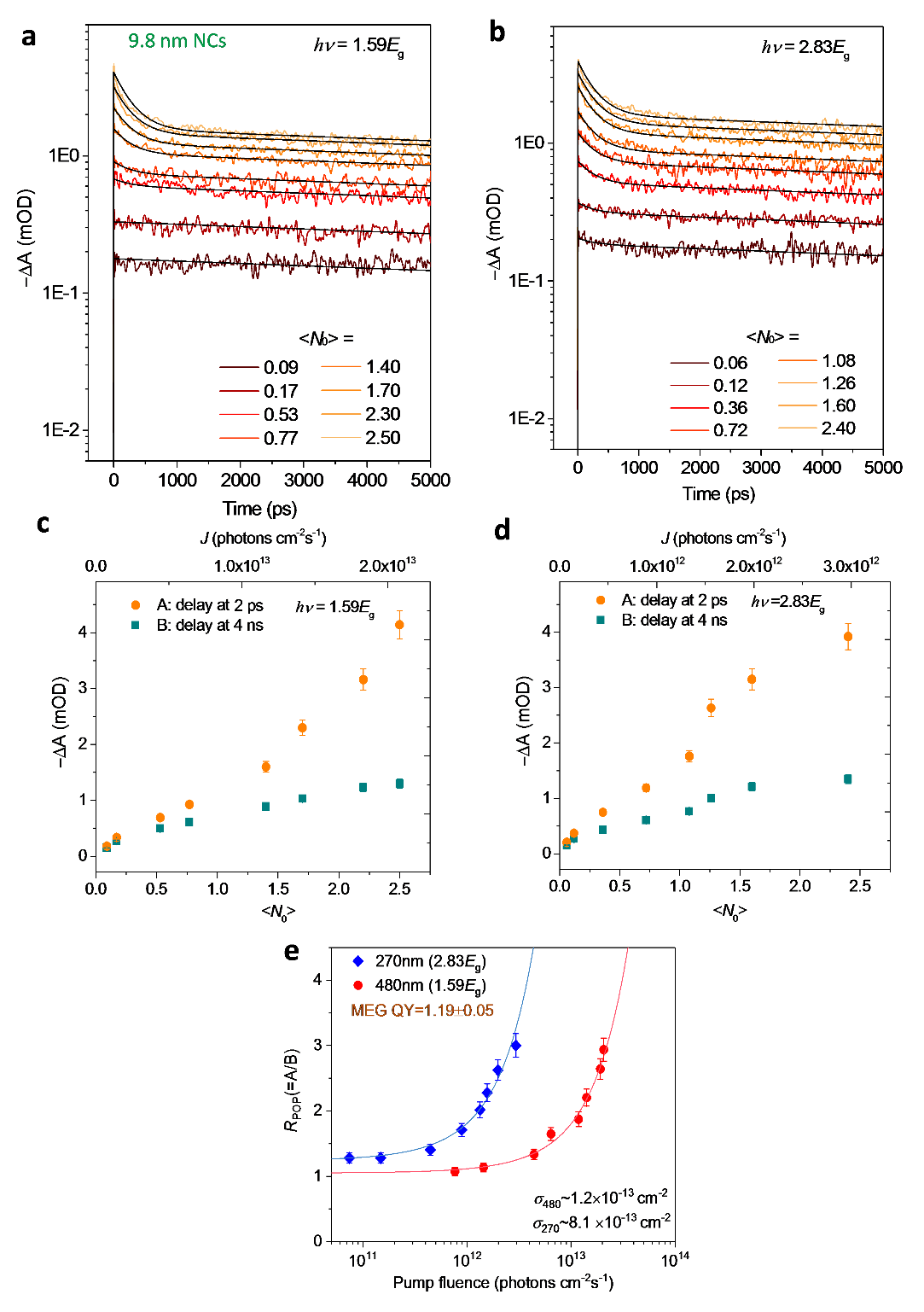


**Supplementary Figure 6** Band-edge PB dynamics at different <*N*_0_> generated by photon energies of (a) 1.59*E*g and (b) 2.83*E*g for 9.8-nm sized FAPbI_3_ NCs. Solid black lines are bi-exponential fittings with the fast decay of 275 ± 30 ps for biexciton lifetime. (c)-(d) TA amplitudes at delay times of A(Δt ~ 2ps) and B(Δt ~ 4ns) under different photoexcitations. (e) *R*_POP_ (=A/B) as a function of pump fluence.


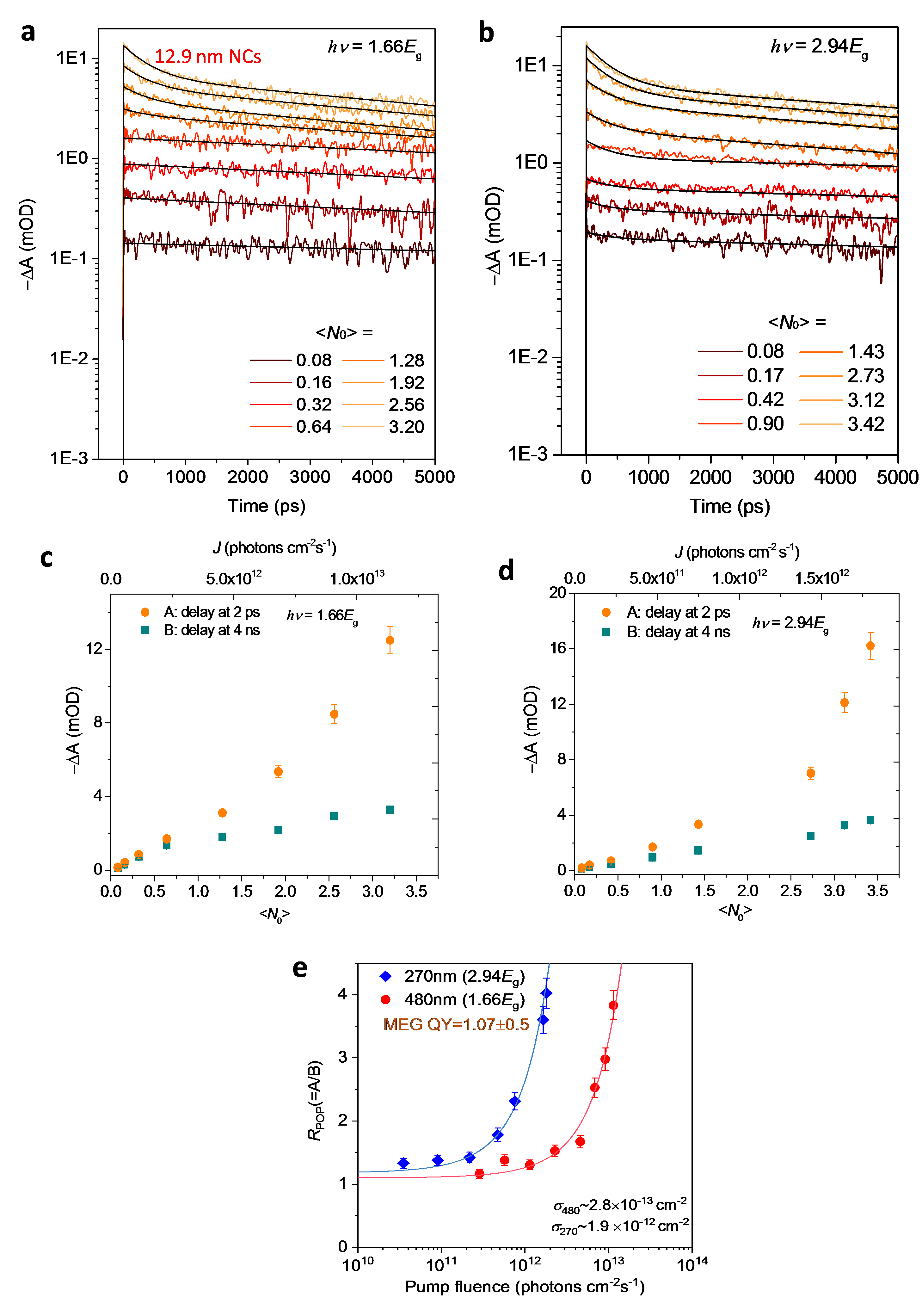


**Supplementary Figure 7** Band-edge PB dynamics at different <*N*_0_> generated by photon energies of (a) 1.66*E*g and (b) 2.94*E*g for 12.9-nm sized FAPbI_3_ NCs. Solid black lines are bi-exponential fittings with the fast decay of 400 ± 40 ps for biexciton lifetime. (c)-(d) TA amplitudes at delay times of A(Δt ~ 2ps) and B(Δt ~ 4ns) under different photoexcitations. (e) *R*_POP_ (=A/B) as a function of pump fluence.


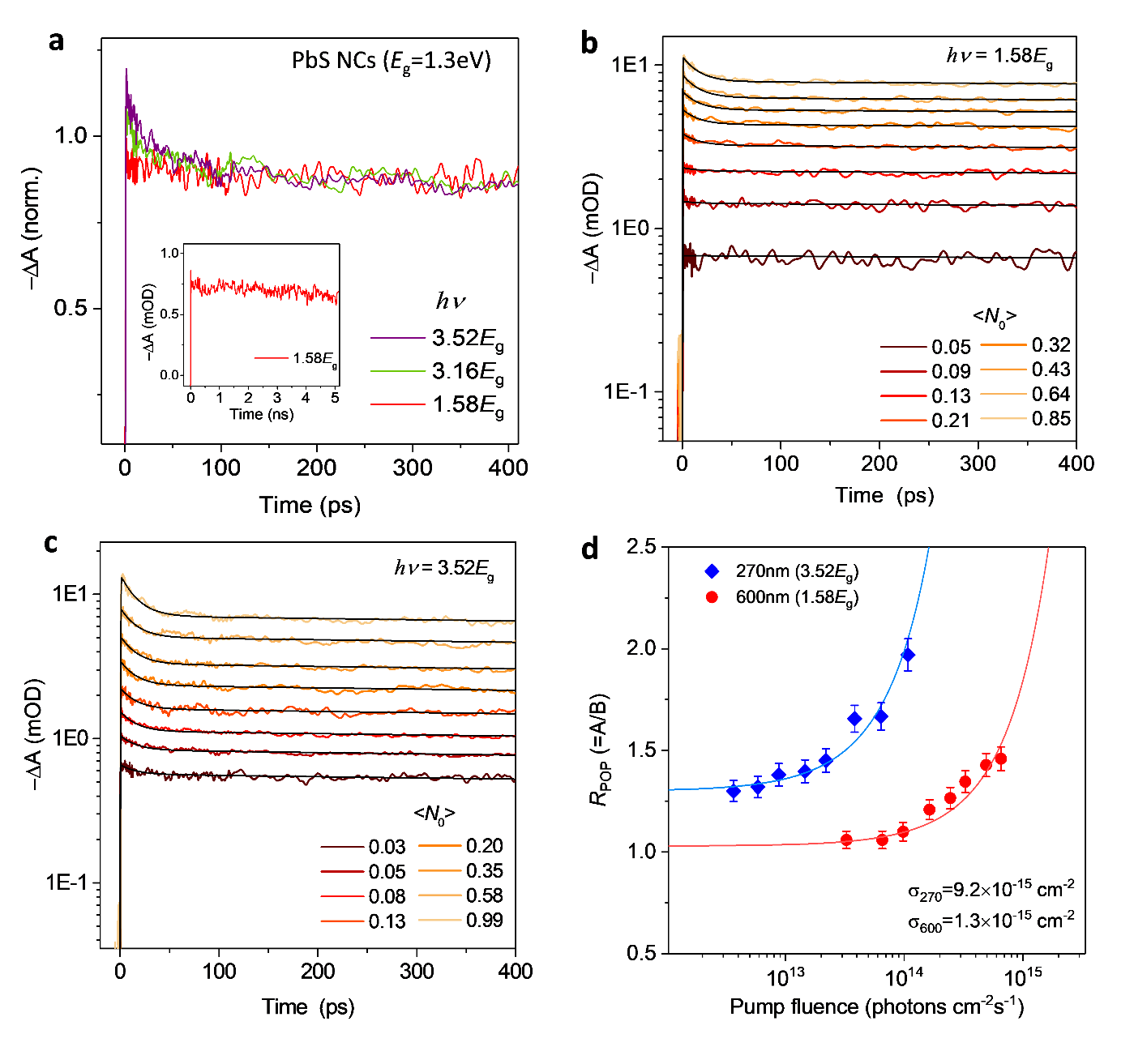


**Supplementary Figure 8** (a) Normalized PB dynamics probed at 1Se-1Sh transition (1.3eV) of PbS NCs (diameter ~3.5 nm with <*N*_0_>=0.05. Inset shows the PB dynamics at 1.58*E*_g_ excitation over a longer time window. PB dynamics at different <*N*_0_> generated by photon energy of (b) 1.58*E*_g_ and (c) 3.52*E*_g_. Solid lines in (b)-(c) are bi-exponential decay fittings with the fast decay of 16±2ps for biexciton lifetime. (d) *R*_POP_ determined by the ratio of PB amplitude at A(Δt ~ 1ps) and B(Δt ~300 ps) as a function of pump fluence. Inset shows fitted absorption cross-sections.


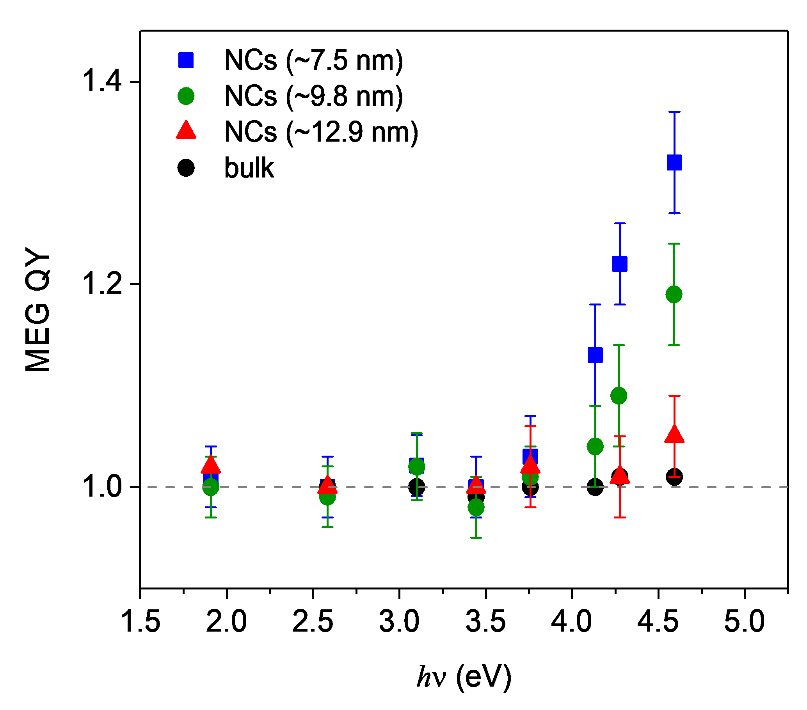


**Supplementary Figure 9** MEG quantum yield (QY) as a function of pump photon energies of FAPbI_3_ NCs with different sizes (with edge length labeled in the brackets) and their bulk-film counterpart.


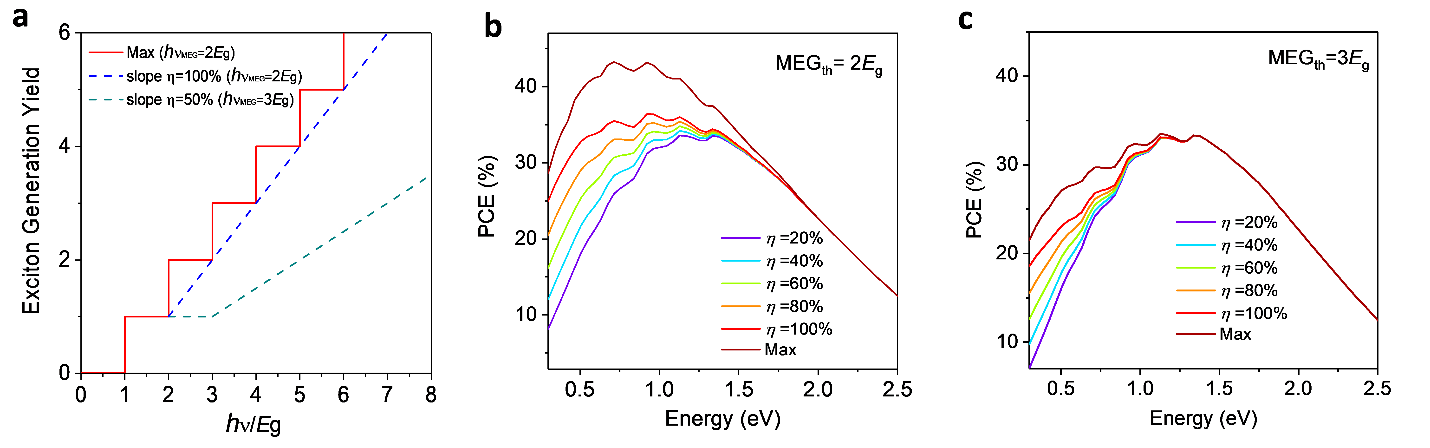


**Supplementary Figure 10** (a), multiple exciton generation yield as a function of *hv*/*E*g for an ideal case (max, solid line) and two non-ideal cases as examples (dashed lines). Calculated PCE under AM1.5 solar illumination as a function of *E*_g_ for MEG threshold of (b) 2*E*_g_ and (c) 3*E*_g_ at different MEG efficiencies. The details of the calculation are given in Supplementary Note 3.


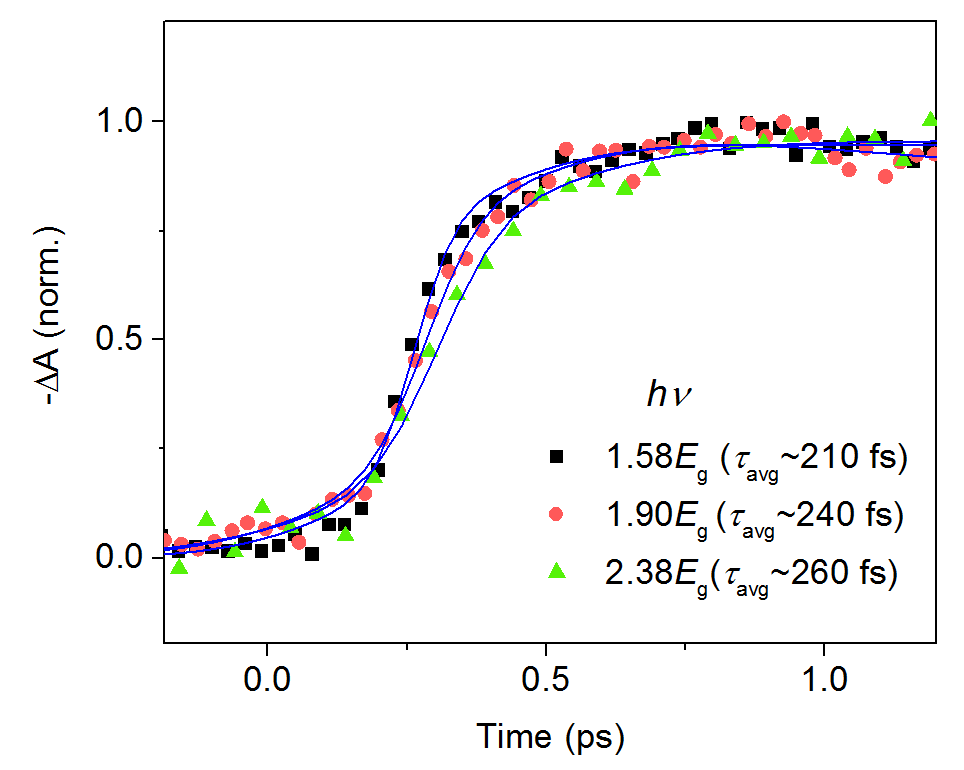


**Supplementary Figure 11** Normalized PB build up dynamics probed at 1Se-1Sh transition (1.3eV) of PbS NCs (diameter ~3.5 nm) at <*N*_0_>=0.02. Solid blue lines are bi-exponential fits. The average rise time *τ*_avg_ is an amplitude averaged lifetime. The fast rise originates from the build-up of the 1p states and slower rise from the 1p-1s relaxation^1^, which is more obvious in strongly-confined PbS NCs compared to the single exponential rise for FAPbI_3_ NCs in Fig. 4b in main text.


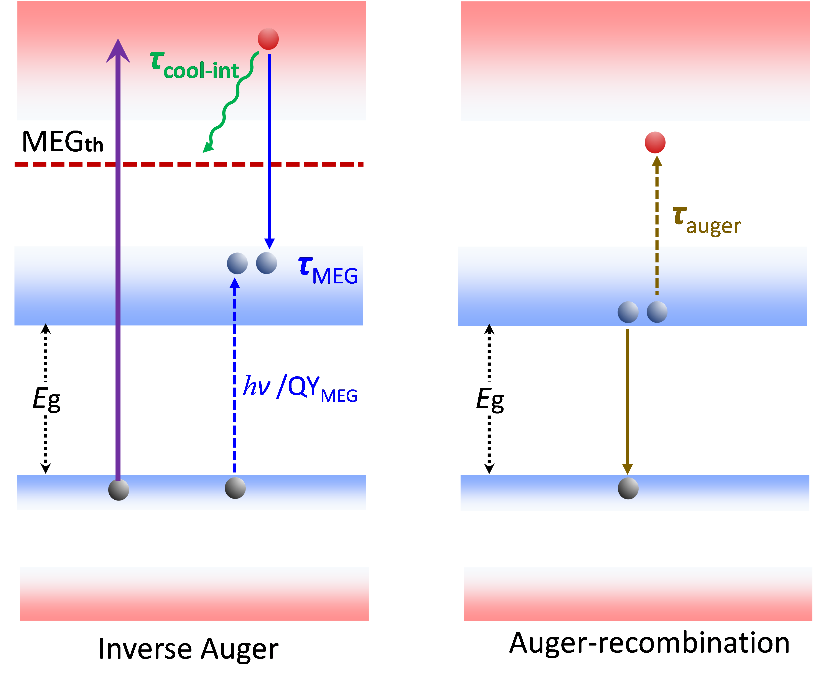


**Supplementary Figure 12** Comparison of the inverse Auger mechanism (left) for MEG and the Auger recombination mechanism (right) for hot-carrier cooling. In the Auger-recombination process, a carrier in one of the electron-hole pairs can be re-excited to higher energy levels from the recombination of second electron-hole pair. Consequently, this induces a longer hot-carrier cooling lifetime. This process is also known as Auger-heating.^2, 3^


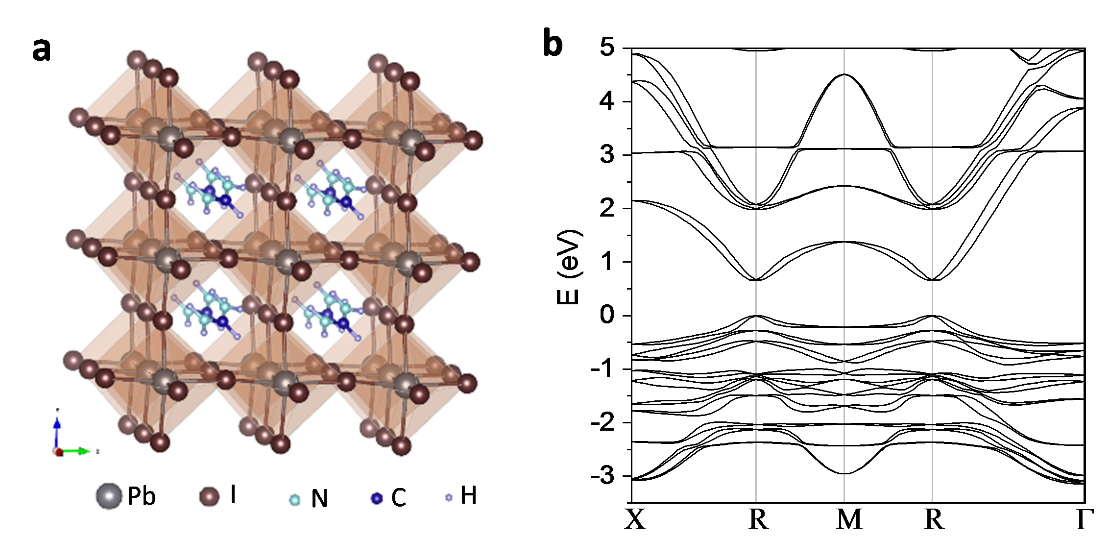


**Supplementary Figure 13** (a), Optimized cubic structure of FAPbI_3_. (b), DFT calculated band structure of FAPbI_3_.

**Supplementary Table 1** Emission and TA peak positions of FAPbI_3_ NCs and bulk-film.

| Sample  Name | Ligand  Used* | Centrifuge Speed (rpm) | Average edge length  (nm) | Emission peak position  (nm) | TA peak position (nm) |
| --- | --- | --- | --- | --- | --- |
| Batch-1 | long (OA) | 11,000 | 7.5±1.2 | 746±2 | 727±3 |
| Batch-2 | long (OA) | 7,000 | 8.0±1.0 | 755±2 | 747±3 |
| Batch-3 | long (OA) | 3,000 | 9.8±0.5 | 772±2 | 764±2 |
| Batch-4 | short (PA) | 5,000 | 11.5±1.0 | 786±2 | 778±2 |
| Batch-5 | short (PA) | 1,000 | 12.9±0.5 | 800±2 | 795±1 |
| Bulk-film | - | - | - | 807±2 | 805±1 |

*OA=oleate, PA= propionate

**Supplementary Table 2** Experimentally measured MEG threshold and slope efficiency of various NCs in solution.

| NCs | Threshold  (*hv/E*_g_) | Slope efficiency | Stirring? | Technique | Year | Refs |
| --- | --- | --- | --- | --- | --- | --- |
| FAPbI_3_ | 2.25-2.5 | 0.75 | Y | TA | 2018 | this work |
| PbS | ~3 | 0.4 |  |  |  |  |
| PbS | ~3 | 0.26-0.4 | Y | TA | 2013 | ^4^ |
| PbSe | ~3 | 0.4 |  |  |  |  |
| PbSe | ~3 | 0.4 | Y | TA | 2010 | ^5^ |
| InP | ~2.1 | 0.31 | Y | TA | 2010 | ^6^ |
| PbSe | ~3 | 0.4 | Y | TA, TRPL | 2008 | ^7^ |
| InAs | 2-2.5 | 0.35 | Y | TA | 2007 | ^8^ |
| PbSe/CdSe  Core/Shell | ~2.2 | 0.25 | Y | TRPL | 2014 | ^9^ |
| It is highly possible that the early works listed below could have overestimated the MEG yield (refer to the details in Ref ^7^) | | | | | | |
| Si | 2.4 | >0.9 | N | TA | 2007 | ^10^ |
| CdSe | 2.5 | 1.12 | N | TA | 2005 | ^11^ |
| PbSe | 2.9 | 1.14 | N |  |  |  |
| PbSe, PbS | ~2.5-3.5 | >0.9 | N | TA | 2004 | ^12^ |

**Supplementary Note 1. Exciton Bohr diameter.**

Based on the effective-mass approximation (EMA), the energy of an 1s-1s electron-hole pair state in a spherical quantum confined NC can be expressed by^13^

$E_{1s1s}\left( D \right)=E_{g,bulk}+\frac{{\pi^{2}e}^{2}D_{x}}{\varepsilon D^{2}}-3.572\frac{e^{2}}{\varepsilon D}$ (S1)

Where *D* is the diameter of NC, *E*_g,bulk_ the bulk bandgap energy, *ɛ* the effective dielectric constant. The second term is the carrier kinetic energy contribution, while the third term is electron-hole Coulomb interaction energy. We obtained the FAPbI_3_ exciton Bohr diameter *D*_X_ by fitting the size-dependent band-edge energies in Fig. 1b in main text using Eqn. S1, with *E*_g,bulk_~1.5eV from FAPbI_3_ single crystal,^14^ and *ɛ*_eff_ is ~11.4.^15^ The fitted exciton Bohr diameter is *D*_X_=12.3±0.2 nm. Given that the ~7.5-13 nm edge length (*L*) for our FAPbI_3_NCs, *D*_X_/*L* is in the region of ~1.64-0.95, corresponding to the intermediate confinement (*D*_X_/*L*>1) regime to weak confinement (*D*_X_/*L*<1). The small binding energies (~14-25 meV for our large to small FAPbI_3_ NCs) is negligible compared to the exciton energy. Here, we simply used the exciton energy (determined by TA spectra shown in Supplementary Fig. 3) as the bandgap energy.

**Supplementary Note 2. Absorption cross section**

From Eqn. (1) in the main text, it follows that the fitted $\sigma_{P}$ increases almost linearly with FAPbI_3_ NC volume from 5.7×10^-14^ cm^-2^ to 2.8 ×10^-13^ cm^-2^ at pump wavelength of ~ 480 nm for NCs with sizes ranging 7.5 to 12.9 nm (see insets of Supplementary Figs. 5e-7e). The reported $\sigma_{P}$ at wavelength of ~ 400 nm for 13 nm FaPbI_3_ NCs is approximately 5.2 ×10^-13^ cm^-2^ as determined from TRPL,^16^ which is comparable to our measurements for NCs of similar size. These results thus further validate the accuracy of our method for determining <*N*_0_>.

**Supplementary Note 3. PCE calculation of a single junction solar cell with MEG**

The model for a single junction MEG solar cell can be referred as the detailed balance model introduced by Shockley and Quisser.^17, 18^ The external current $j_{\mathrm{ext}}$ is determined from the difference between the absorbed spectral photon flux $j_{\mathrm{abs}}$ of the AM 1.5G solar irradiance and the emitted photon flux $j_{\mathrm{em}}$at external voltage *V*:

$j_{\mathrm{ext}}\left( E_{g}, V \right)=\int_{E_{g}}^{\infty} dE[j_{\mathrm{abs}}\left( E_{g} \right)-j_{\mathrm{em}}\left( E_{g}, V \right)]$ (S2)

The absorbed photon flux can be expressed as:

$j_{\mathrm{abs}}\left( E \right)=\frac{q\lambda\beta}{hc}AM1.5G$ (S3)

where *q* is the charge unit, $\lambda$the light wavelength, *h* the Plank constant, *c* the speed of light, $\beta$ the multiple exciton generation yield, *E*_th_ the MEG threshold. For the ideal MEG process, *β* follows the stair function, i.e., when the photon energy reaches the N × *E*g, maximum MEG yield is N (solid line in Supplementary Fig. 10a). For the non-ideal case (dashed lines in Supplementary Fig. 10a), as in our experiment, *β* increases linearly with $h\nu$/*E*g, the slope represents the MEG slope efficiency ($\eta_{\mathrm{MEG}}$).

The emitted photon flux for a flat geometry is calculated based on the generalized Planck’s law:

$j_{\mathrm{em}}\left( E,V \right)=\frac{q2\pi E^{2}}{h^{3}c^{2}}\frac{1}{[\exp\left( \frac{E-qV}{\kappa_{B} T} \right)-1]}$ (S4)

where $\kappa_{B}$ is the Boltzmann constant. The PCE limit is calculated as follows:

$PCE=V\times\frac{j_{\mathrm{ext}}\left( E_{g},V \right)}{P_{s}}$ (S6)

where $P_{s}$ is the incident power from AM 1.5G solar irradiation.

**Supplementary References**

1. Schaller, R.D., Agranovich, V.M. & Klimov, V.I. High-efficiency carrier multiplication through direct photogeneration of multi-excitons via virtual single-exciton states. *Nat. Phys.* **1**, 189 (2005).

2. Li, M.J. et al. Slow cooling and highly efficient extraction of hot carriers in colloidal perovskite nanocrystals. *Nat. Commun.* **8**, 14350 (2017).

3. Fu, J.H. et al. Hot carrier cooling mechanisms in halide perovskites. *Nat. Commun.* **8**, 1300(2017).

4. Midgett, A.G. et al. Size and Composition Dependent Multiple Exciton Generation Efficiency in PbS, PbSe, and PbSxSe1-x Alloyed Quantum Dots. *Nano Lett* **13**, 3078-3085 (2013).

5. Midgett, A.G., Hillhouse, H.W., Hughes, B.K., Nozik, A.J. & Beard, M.C. Flowing versus Static Conditions for Measuring Multiple Exciton Generation in PbSe Quantum Dots. *J. Phys. Chem. C* **114**, 17486-17500 (2010).

6. Stubbs, S.K. et al. Efficient carrier multiplication in InP nanoparticles. *Phys. Rev. B* **81**, 081303 (2010).

7. Mcguire, J.A., Joo, J., Pietryga, J.M., Schaller, R.D. & Klimov, V.I. New Aspects of Carrier Multiplication in Semiconductor Nanocrystals. *Accounts Chem. Res.* **41**, 1810-1819 (2008).

8. Schaller, R.D., Pietryga, J.M. & Klimov, V.I. Carrier multiplication in InAs nanocrystal quantum dots with an onset defined by the energy conservation limit. *Nano Lett* **7**, 3469-3476 (2007).

9. Cirloganu, C.M. et al. Enhanced carrier multiplication in engineered quasi-type-II quantum dots. *Nat. Commun.* **5**, 4148 (2014).

10. Beard, M.C. et al. Multiple exciton generation in colloidal silicon nanocrystals. *Nano Lett.* **7**, 2506-2512 (2007).

11. Schaller, R.D., Petruska, M.A. & Klimov, V.I. Effect of electronic structure on carrier multiplication efficiency: Comparative study of PbSe and CdSe nanocrystals. *Appl. Phy. Lett.* **87**, 253102 (2005).

12. Ellingson, R.J. et al. Highly efficient multiple exciton generation in colloidal PbSe and PbS quantum dots. *Nano Lett.* **5**, 865-871 (2005).

13. Gaponenko, S.V. Optical Properties of Semiconductor Nanocrystals. (Cambridge University Press, Cambridge; 1998).

14. Han, Q. et al. Single Crystal Formamidinium Lead Iodide (FAPbI3): Insight into the Structural, Optical, and Electrical Properties. *Adv. Mater.* **28**, 2253-2258 (2016).

15. Galkowski, K. et al. Determination of the exciton binding energy and effective masses for methylammonium and formamidinium lead tri-halide perovskite semiconductors. *Energ Environ. Sci.* **9**, 962-970 (2016).

16. Fang, H.H. et al. Exciton Recombination in Formamidinium Lead Triiodide: Nanocrystals versus Thin Films. *Small* **13**, 1700673 (2017).

17. Shockley, W. & Queisser, H.J. Detailed balance limit of efficiency of p‐n junction solar cells. *J. Appl. Phys.* **32**, 510-519 (1961).

18. Rühle, S. Tabulated values of the Shockley–Queisser limit for single junction solar cells. *Solar Energy* **130**, 139-147 (2016).
